# New Alkaloids and Polyketides from the Marine Sponge-Derived Fungus *Penicillium* sp. SCSIO41015

**DOI:** 10.3390/md17070398

**Published:** 2019-07-05

**Authors:** Xiaoyan Pang, Guodi Cai, Xiuping Lin, Limbadri Salendra, Xuefeng Zhou, Bin Yang, Junjian Wang, Junfeng Wang, Shihai Xu, Yonghong Liu

**Affiliations:** 1 College of Chemistry and Materials Science, Jinan University, Guangzhou 510632, China; 2 CAS Key Laboratory of Tropical Marine Bio-resources and Ecology/Guangdong Key Laboratory of Marine Materia Medica, South China Sea Institute of Oceanology, Chinese Academy of Sciences, Guangzhou 510301, China; 3 College of Pharmacy, Jinan University, Guangzhou 510632, China; 4 School of Pharmaceutical Sciences, Sun Yat-Sen University, Guangzhou 510006, China

**Keywords:** sponge-derived fungus, *Penicillium* sp., alkaloid, X-ray single crystal diffraction, antibacterial, cytotoxic activity

## Abstract

The sponge-derived fungus *Penicillium* sp. SCSIO41015 cultured on solid rice medium yielded twenty-one compounds (**1**–**21**), including two new alkaloids (**1** and **2**) and one new pyrone derivative (**3**). Their structures were elucidated by analysis of 1D/2D NMR data and HR–ESI–MS. Their absolute configurations were established by single-crystal X-ray diffraction analysis and comparison of the experimental with reported specific rotation values. Compound **16** exhibited selective cytotoxic activity against the human gastric cancer cells MGC803, with IC_50_ value of 5.19 μM. Compounds **9** and **18** showed weak antibacterial activity against *Staphylococcus aureus* and *Acinetobacter baumannii,* respectively, both with MIC values of 57 μg/mL. Furthermore, compound **16** displayed potent antibacterial activity against *S. aureus* with an MIC value of 3.75 μg/mL.

## 1. Introduction

**Figure 1:**
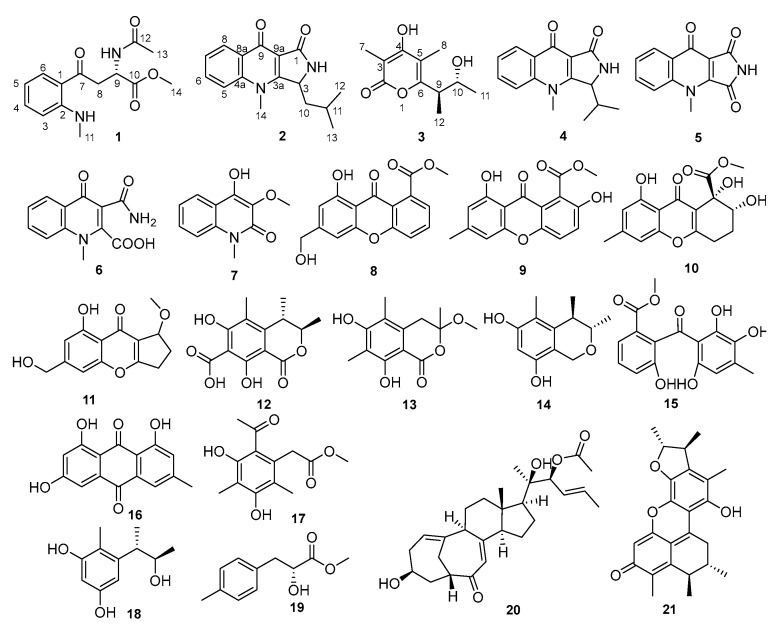
Chemical structures of compounds **1**–**21**.

A considerable number of structurally unique secondary metabolites with biological and pharmacological activities have been isolated from the marine-derived fungi in recent years [1,2]. Marine-derived *Penicillium* and *Aspergillus* are reported to be the most ubiquitous genera [1]. *Penicillium* has become one of the most attractive fungal genera to search for bioactive molecules, since the advent of penicillin [3]. More than 400 new marine natural products, including half the number of polyketides and a quarter the number of nitrogen compounds have been isolated from marine-derived *Penicillium* fungi, and 58% of the new products have displayed bioactivities such as anticancer, antibacterial, anti-HIV, and anti-inflammatory properties [4]. As part of our continued searching for bioactive secondary metabolites from sponge-derived fungi [5,6,7], the fungus *Penicillium* sp. SCSIO41015 was investigated. Three new compounds (**1**–**3**) and eighteen known ones (**4**–**21**) were isolated from the rice solid culture extract of the fungus *Penicillium* sp. SCSIO41015 shown in Figure 1. Nearly all compounds were evaluated on their cytotoxic activities against six human cancer cells and their antibacterial activities against five pathogenic bacteria. Herein, we described the isolation, structure elucidation, and bioactivity evaluation of the twenty-one compounds, as well as the potential biosynthetic pathway of these alkaloids.

## 2. Results and Discussion

### 2.1. Isolation and Structural Elucidation

**Figure 2:**
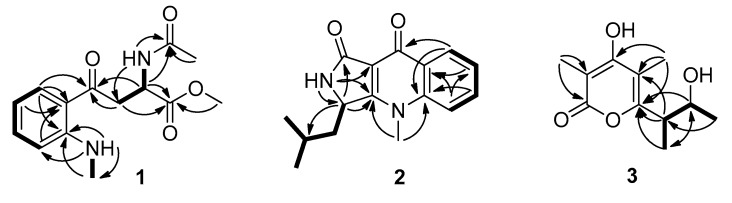
Correlation spectroscopy (COSY) and key heteronuclear multiple bond correlation (HMBC) correlations of compounds **1**–**3**.

Compound **1** was obtained as a colorless oil. Its molecular formula was established as C_14_H_1__8_N_2_O_4_, through high resolution electrospray ionization mass spectroscopy (HR–ESI–MS) [M + H]^+^ peak at m/z 279.1336 (calculated forC_14_H_1__9_N_2_O_4_, 279.1339),indicating seven degrees of unsaturation. Its ^1^H NMR data (Table 1) showed four aromatic protons (*δ*_H_ 7.81, dd, *J* = 7.7, 1.4 Hz, H-6; 7.43, td, *J* = 7.7, 0.7 Hz, H-4; 6.74, d, *J* = 8.4 Hz, H-3; and 6.60, td, *J* = 7.7, 0.7 Hz, H-5), one methine (*δ*_H_ 4.76, td, *J* = 7.0, 5.6 Hz, H-9), one methylene (*δ*_H_ 3.44, dd, *J* = 17.5, 7.0 Hz, H-8a; 3.40, dd, *J* = 17.5, 5.6 Hz, H-8b), three methyls (*δ*_H_ 3.61, s, H_3_-14; 2.85, d, *J* = 4.9 Hz, H_3_-11; 1.82, s, H_3_-13), and two active hydrogen protons (*δ*_H_ 8.59, dd, *J* = 9.1, 4.2 Hz, NH-11; 8.23, d, *J* = 7.7 Hz, NH-9). Accordingly, the ^13^C NMR (DEPT) (Table 1) data also displayed fourteen carbon signals, including four sp^2^methines, a sp^3^methylene (*δ*_C_ 40.2, C-8), a sp^3^methine (*δ*_C_ 47.9, C-9), three methyls (*δ*_C_ 52.0, C-14; 29.0, C-11; 22.3, C-13), two sp^2^ non-protonated carbons, and three carbonyls (*δ*_C_ 198.1, C-7; 172.2, C-10; 169.2, C-12). Part of its NMR data were similar to *N*-acetyl-6-nitrotryptophanmethyl ester synthetized by R.R. King [8]. The obvious differences were that the signals of a nitro group and a double-bond were absentin compound **1,** while an aromatic proton, and a methyl and a carbonyl group were observed. The changes indicated that the five-membered ring opened between C-2 and C-3 in *N*-acetyl-6-nitrotryptophanmethyl ester, which were confirmed by the ^1^H-^1^H correlation spectroscopy (COSY) of H-3/H-4/H-5/H-6, H_3_-11/NH-11, and the heteronuclear multiple bond correlation (HMBC) spectrum of H_2_-8, H-9, and H-6, to C-7 (Figure 2). Thus, the planar structure of **1** was further ascertained by the HMBC spectrum (Figure 2). The potential biosynthetic pathway of **1** was speculated that **1** was synthesized from *l*-tryptophan as shown in Figure S1. Combining the comparison experimental specific rotation value ${\left[\alpha \right]}_{\text{D}}^{20}$ (+58.1, CHCl_3_) of compound **1** with ${\left[\alpha \right]}_{\text{D}}^{20}$ (+141, CHCl_3_) of the reported (2*S*)-methyl 2-acetamido-4-(2’-acetamidophenyl)-4-oxobutanoate [9], the absolute configuration of **1** was established to be *S*. Therefore, compound **1** was named (*S*)-methyl 2-acetamido-4-(2-(methylamino)phenyl)-4-oxobutanoate.

**Figure 3:**
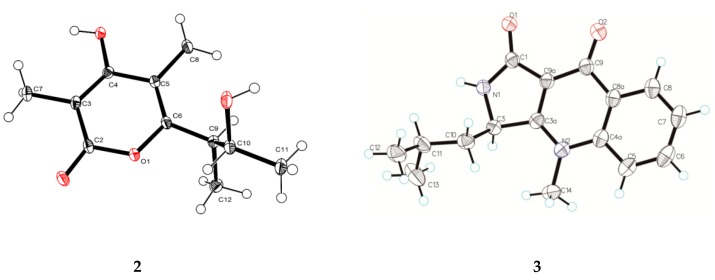
ORTEP drawings of compounds **2** and **3**.

Compound **2** was isolated as a light-yellow powder that returned a HR–ESI–MS pseudomolecularion (*m*/*z* 293.1263, [M + Na]^+^), corresponding to a molecular formula (C_16_H_1__8_N_2_O_2_) requiring nine degrees of unsaturation. Analysis of its NMR data (Table 1) revealed that the structural features of **2** closely resembled those of quinolactacin D [10]. The only difference was a methine (*δ*_H_ 4.86, brd, *J* = 8.4 Hz, *δ*_C_ 52.5, CH-3) in **2** instead of the oxygenated non-protonated carbon in quinolactacin D, which was confirmed by the correlations of H-3 to C-1 (*δ*_C_ 167.9), C-3a (*δ*_C_ 165.6), C-10 (*δ*_C_ 42.3), C-11 (*δ*_C_ 24.7) in the HMBC spectrum and the COSY cross-peaks of H-3/H-10a/H-10b (Figure 2). Therefore, **2** was a *N*-methyl quinolone lactam, and the planar structure of **2** was determined as hydroxyl at C-3 reductive quinolactacin D, based on 2D NMR data (Figure 2). The deduction above was supported by the Cu K*α* single crystal X-ray diffraction analysis (ORTEP, oak ridge thermal ellipsoid plot, drawing in Figure 3), while the X-ray data showed that the crystal of **2** existed as a pair of enantiomers. The chiral phase of HPLC analysis showed that were two conjoint peaks that could not separate well, also **2** dissolved in a solvent that was unstable and then changed to quinolactacin D, as speculated in the reference [10]. Compound **2** appeared to be synthesized from kynurenine and leucine, which was similar to the speculation reported by Nakagawa et al. [11]; shown in Figure S1. Thereby, **2** was elucidated as a pair of enantiomers and named quinolactacin E.

The HR–ESI–MS data showed the molecular formula of compound **3** to be C_11_H_16_O_4_, which indicated four degrees of unsaturation in this molecular structure. The ^1^H NMR and ^13^C NMR data (Table 1) of **3** were quite similar with those of germicidin L [12], except for the absence of an aromatic proton signal, which was replaced by a non-protonated carbon (*δ*_C_ 110.0, C-5) and amore methyl group (*δ*_H_ 2.01, s, *δ*_C_ 10.0, CH_3_-8). The changes were confirmed by the HMBC correlations of H_3_-8 to C-4 (*δ*_C_ 168.0), C-5, C-6 (*δ*_C_ 161.6), indicating **3** was methyl-substituted germicidin L at position of C-5. The proposed structure was further verified by X-ray diffraction analysis of the single crystal of compound **3** (ORTEP drawing in Figure 3). Its single-crystal X-ray diffraction experiment with Cu Kα radiation and the value of the Flack parameter, 0.07(7), allowed the assignment of the absolute configuration of **3** as 9*R*, 10*R*. Thus, compound **3** was established as 4-hydroxy-6-((2*R*,3*R*)3-hydroxybutan-2-yl)-3,5-dimethyl-2*H*-pyran-2-one and named germicidin O.

Polyketides and alkaloids are two major secondary metabolites from marine-derived fungi, and the same polyketides or alkaloids were often isolated from different fungi. According to the detailed spectroscopic analysis and comparison to the reported literature data, the known compounds (**4**–**21**) were identified as quinolactacin B (**4**) [10], quinolonimide (**5**) [10], quinolonic acid (**6**) [10], 4-hydroxy-3-methyl-2(1*H*)-quinolinone (**7**) [13], sydowinin A (**8**) [14], pinselin (**9**) [15], *β*-diversonolic ester (**10**) [16], coniochaetone J (**11**) [17], dihydrocitrinone (**12**) [18], stoloniferol A (**13**) [18], 6,8-dihydroxy-3,4,5-trimethylisochroman (**14**) [19], moniliphenone (**15**) [20], frangula-emodin (**16**) [21], methyl-2-(2-acetyl-3,5-dihydroxy-4,6-dimethylphenyl) acetate (**17**) [22], phenol A (**18**) [23], latifolicinin C (**19**) [24], penicitrinone A (**20**) [25], and 22-acetylisocyclocitrinol A (**21**) [26], respectively.

### 2.2. Biological Activity

Compounds **1**–**21**, excepting **2** (since it was unstable in solvent and its quality was poor after the second preparation) were evaluated on their cytotoxic activities against human gastric cancer cells MGC803, human breast cancer cells MDA-MB-231, human prostate cancer cells C4-2B, human osteosarcoma 143B, and human hung cancer cells A549, using the CCK-8 method. Compound **16** selectively exhibited cytotoxic activity against MGC803 with IC_50_ value of 5.19 μM. The antibacterial activities of all compounds, except **2**, were estimated against *A.baumannii* (ATCC 19606), *Klebsiella pneumonia* (ATCC 13883), *Escherichia coli* (ATCC 25922), *S.aureus* (ATCC 29213) and *Enterococcus faecalis* (ATCC 29212). Compounds **9**, **16**, **18**, and **21** with 100 μg/disc showed an inhibition zone against *S. aureus* with a diameter of about 9, 12, 21, and 7 mm, respectively. Compounds **18** and **13** with 100 μg/disc displayed an inhibition zone against *A. baumannii* both with a diameter of about 8 mm (Figure S2). Further, their minimum inhibitory concentrations (MIC) were tested. The MIC values of compounds **9** and **16** against *S. aureus* were 57 and 3.75 μg/mL, respectively, and the MIC value of compound **18** against *A. baumannii* was 57 μg/mL, while the other compounds showed more than 100 μg/mL. Ampicillin and gentamicin were used as positive control against *S. aureus* and *A. baumannii*, respectively, with the MIC values of 0.45 and 0.9 μg/mL (Table S1). 

## 3. Materials and Methods 

### 3.1. General Experimental Procedures

UV spectra were recorded on a UV-2600 UV-Vis spectrophotometer (Shimadzu, Japan). Optical rotations were measured using a MCP-500 polarimeter (Anton, Austria). 1D and 2D NMR spectra were measured on a Bruker Avance 500 MHz or 700 MHz NMR spectrometer (Fällanden, Switzerland) with TMS as an internal standard. HR–ESI–MS data were recorded on a maXis Q-TOF mass spectrometer in the positive ion mode (Bruker, Fällanden, Switzerland). X-ray diffraction intensity data were collected on XtalLAB PRO single-crystal diffractometer, using Cu Kα radiation (Rigaku, Japan). The data were corrected for absorption, using CrysAlisPro 1.171.39.33c (Rigaku Oxford Diffraction, 2017). The structures were solved by the direct methods (SHELXS 97), expanded using difference Fourier techniques, and refined by full-matrix least-squares calculation. All H atoms were fixed at the calculated positions and the non-hydrogen atoms were refined anisotropically. HPLC was performed on the Hitachi Primaide with the YMC ODS SERIES column (YMC-Pack ODS-A, YMC Co. Ltd., Kyoto, 250 × 10 mm I.D., S-5 μm, 12 nm). Column chromatography (CC) was carried out on silica gel (200–300 mesh, Jiangyou Silica Gel Development Co., Yantai, China), YMC Gel ODS-A (12 nm, S-50 μm YMC, MA, USA), and Sephadex LH-20 (40–70 μm, Amersham Pharmacia Biotech AB, Uppsala, Sweden). Spots on the thin-layer chromatography (TLC) plates were analyzed under UV light, or through heating, after spraying with amixed solvent of saturated vanillin and 5% H_2_SO_4_ in H_2_O. The TLC plates with silica gel GF254 (0.4–0.5 mm, Qingdao Marine Chemical Factory, Qingdao, China) were used for the analyses and preparations.

### 3.2. Fungal Material

The fungal strain SCSIO41015 was obtained from a *Callyspongia* sp. sponge, which was collected from the sea area near Xuwen County, Guangdong Province, China. The strain SCSIO41015 was stored on MB agar (malt extract 15 g, agar 16 g, sea salt 10 g, water 1 L, pH 7.4–7.8) slants at 4 °C and deposited at the Key Laboratory of Tropical Marine Bio-Resources and Ecology, Chinese Academy of Science. The ITS1-5.8S-ITS2 sequence region (508 base pairs, GenBank accession No. MK813890) of strain SCSIO41015 was amplified by PCR, and DNA sequencing showed that it shared significant homology with several species of *Penicillium*. The 508 base pair ITS sequence had 99% sequence identity to that of the *Penicillium citrinum* strain SCSGAF0167 (GenBank accession No. JN851046.1). Then, it was designated as a member of *Penicillium* sp. and named *Penicillium* sp. SCSIO41015.

### 3.3. Fermentation and Extraction

The mass fermentation of this fungus was carried out at 25 °C for 30 days, using a rice medium in a 1 L flask (×45) and every flask contained 200 g of rice, 2.5 g of sea salt, and 200 mL of tap H_2_O. The flasks were cultivated statically at 25 °C, under normal day–night cycle. After 30 days, the cultures were soaked in acetone (500 mL/flask) and mashed into small pieces and sonicated for 20 min. Then, the acetone was evaporated under reduced pressure to obtain an aqueous solution, which was extracted with ethyl acetate (EtOAc), for three times. The rice residue was also extracted with EtOAc. Both of the EtOAc solutions were concentrated under reduced pressure to gain a crude extract. The crude extract was suspended in MeOH and then partitioned with an equal volume of petroleum ether to remove the oil. Lastly, the MeOH solution was concentrated under reduced pressure to afford a brown extract (77.0 g).

### 3.4. Isolation and Purification

The brown extract was subjected to silica gel CC, which was eluted with CH_2_Cl_2_ and MeOH mixed solvent in a step gradient (100:0–5:1, *v*/*v*) and separated into eight fractions (Fr-1–Fr-8). Fr-1 (1.4 g) was applied to a Sephadex LH-20 column eluted with MeOH and reversed-phase C-18 MPLC with MeOH/H_2_O (10:90–100:0, *v*/*v*) to gain four sub-fractions (Fr-1-1–Fr-1-4). Fr-1-1 was further purified with semi-preparative HPLC (60% CH_3_CN/H_2_O, 2 mL/min) to afford **8** (8.3 mg, *t_R_* = 10.0 min), **9** (6.4 mg, *t_R_* = 14.5 min), and **16** (3.8 mg, *t_R_* = 22.4 min). Fr-1-3 was further separated with semi-preparative HPLC (60% CH_3_CN/H_2_O, 2 mL/min) to yield **20** (26.1 mg, *t_R_* = 10.8 min). Fr-1-4 was further purified with semi-preparative HPLC (27% CH_3_CN/H_2_O, 2 mL/min) to gain **12** (18.3 mg, *t_R_* = 19 min). Fr-2 (8.5 g) was subjected to silica gel CC eluted with PE and an acetone mixed solvent in a step gradient (10:1–0:1, *v*/*v*), to get three sub-fractions (Fr-2-1–Fr-2-3). Fr-2-2 was applied to a Sephadex LH-20 column eluted with MeOH and reversed-phase C-18 MPLC with MeOH/H_2_O (10:90–100:0, *v*/*v*), and was further purified with a semi-preparative HPLC to obtain five sub-fractions (Fr-2-2-1–Fr-2-2-5). Fr-2-2-1 was separated with semi-preparative HPLC (24% CH_3_CN/H_2_O, 2 mL/min) to afford **10** (7.6 mg, *t_R_* = 46.0 min), **14** (42.4 mg, *t_R_* = 37.0 min), and **15** (23.0 mg, *t_R_* = 59.0 min). Fr-2-2-4 was further isolated by semi-preparative HPLC (45% CH_3_CN/H_2_O, 2 mL/min) to obtain **21** (8.5 mg, *t_R_* = 27.0 min). Fr-3 (3.9 g) was subjected to a Sephadex LH-20 column eluted with MeOH, and reversed-phase C-18 MPLC with MeOH/H_2_O (10:90–100:0, *v*/*v*) to get two sub-fractions (Fr-3-1–Fr-3-2). Fr-3-1 was further purified with semi-preparative HPLC (35% CH_3_CN/H_2_O, 2 mL/min) to obtain **13** (7.4 mg, *t_R_* = 16.0 min). Compound **7** (21.0 mg, t_R_ = 13.4 min) was obtained from Fr-3-2 with semi-preparative HPLC (30% CH_3_CN/H_2_O, 2 mL/min). Fr-4 (3.1 g) was subjected to a Sephadex LH-20 column eluted with MeOH to get three sub-fractions (Fr-4-1–Fr-4-3). Fr-4-1 was applied to reversed-phase C-18 MPLC with MeOH/H_2_O (10:90–100:0, *v*/*v*) and semi-preparative HPLC (30% CH_3_CN/H_2_O, 2 mL/min) to afford **1** (5.5 mg, *t_R_* = 18.5 min) and **17** (20.2 mg, *t_R_* = 17.0 min). Compound **11** (3.8 mg, *t_R_* = 36 min) was gained from Fr-4-2 with semi-preparative HPLC (40% MeOH/H_2_O, 2 mL/min). Fr-6 (1.8 g) was separated by a Sephadex LH-20 column, eluted with MeOH and reversed-phase C-18 MPLC with MeOH/H_2_O (10:90–100:0, *v*/*v*), to get two sub-fractions. This sub-fraction was further isolated by semi-preparative HPLC (42% MeOH/H_2_O, 2 mL/min) to obtain **3** (15.5 mg, *t_R_* = 17.8 min). The other sub-fraction was also purified by semi-preparative HPLC (17% CH_3_CN/H_2_O, 2 mL/min) to gain **19** (5.1 mg, *t_R_* = 17 min). Fr-7 (2.1 g) was separated by a Sephadex LH-20 column eluted with MeOH, reversed-phase C-18 MPLC with MeOH/H_2_O (10:90–100:0, *v*/*v*) and further isolated using semi-preparative HPLC (22% CH_3_CN/H_2_O, 2 mL/min) to yield **2** (3.1 mg, *t_R_* = 45.0 min), **4** (6.4 mg, *t_R_* = 18.0 min ), and **6** (2.7 mg, *t_R_* = 15.0 min). Fr-8 (1.8 g) was separated by a Sephadex LH-20 column eluted with MeOH, reversed-phase C-18 MPLC with MeOH/H_2_O (10:90–100:0, *v*/*v*), and further purified with semi-preparative HPLC (18% CH_3_CN/H_2_O, 2 mL/min) to yield **5** (3.6 mg, *t_R_* = 11.6 min) and **18** (64.3 mg, *t_R_* = 14.2 min).

### 3.5. Spectral Data

*(S)-methyl 2-acetamido-4-(2-(methylamino)phenyl)-4-oxobutanoate* (**1**)—white powder; ${\left[\alpha \right]}_{\text{D}}^{20}$ +58.1 (*c* 0.1, CHCl_3_); UV (MeOH) *λ*_max_(log *ε*) 383 (3.16), 259 (3.31), 228 (3.79), 202 (3.69) nm, ^1^H NMR (DMSO-*d*_6_, 700 MHz), and ^13^C NMR (DMSO-*d*_6_, 175 MHz), Table 1; HR–ESI–MS *m/z* 301.1155 [M + Na]^+^ (calcd. for C_14_H_18_N_2_NaO_4_, 301.1159) and 279.1336 [M + H]^+^ (calcd. for C_14_H_19_N_2_O_4_, 279.1339).

*Quinolactacin E* (**2**)—light yellow crystal; ${\left[\alpha \right]}_{\text{D}}^{25}$ +1.4 (*c* 0.1, MeOH); UV (MeOH) *λ*_max_(log *ε*) 327 (3.86), 314 (3.90), 256 (4.13), 248 (4.15), 217 (4.29) nm; ^1^H NMR (DMSO-*d*_6_, 700 MHz), and ^13^C NMR (DMSO-*d*_6_, 175 MHz), Table 1;HR–ESI–MS *m/z* 293.1263 [M + Na]^+^ (calcd. for C_16_H_18_N_2_NaO_2_, 293.1260).

*Germicidin O* (**3**)—colorless oil; ${\left[\alpha \right]}_{\text{D}}^{25}$ −58.9 (*c* 0.1, MeOH); UV (MeOH) *λ*_max_(log *ε*) 292 (3.74), 208 (4.03) nm; ^1^H NMR (CD_3_OD, 500 MHz), and ^13^C NMR (CD_3_OD, 125 MHz), Table 1; HR–ESI–MS *m/z* 235.0952 [M + Na]^+^ (calcd. for C_11_H_16_NaO_4_, 235.0941).

### 3.6. X-ray Crystal Structure Analysis

*Crystal data (CCDC No.1912523)* for **2** (moiety formula)—C_1__6_H_1__8_N_2_O_2_ (*MW* = 270.32), clear yellowish yellow needle, crystal size = 0.4 × 0.08 × 0.06 mm^3^, monoclinic, space group P2_1_/n; unit cell dimensions: *a* = 12.8540(2) Å, *b* = 8.79200(10) Å, *c* = 13.6987(2) Å, *V* = 1502.38(4) Å^3^, *Z* = 4, *ρ*_calcd_ = 1.195 g cm^−3^, and *T* = 102(4) K, μ(Cu Kα) = 0.640 mm^−1^. A total of 7071 reflections were measured with 2945 independent reflections (*R*_int_= 0.0264, *R*_sigma_ = 0.0315). Final *R* indices [I>2*σ* (*I*)]: *R*_1_ = 0.0428, w*R*_2_ = 0.1199. Final R indices [all date]: *R*_1_ = 0.0514, w*R*_2_ = 0.1257. Largest diff. peak and hole = 0.20 and −0.24eÅ^−3^.

*Crystal data (CCDC No.1912525)* for **3** (moiety formula)—C_11_H_16_O_4_ (*MW* = 212.24), clear light colorless block, crystal size = 0.2 × 0.15× 0.1 mm^3^, monoclinic, space group P2_1_; unit cell dimensions: *a* = 7.61640 (10) Å, *b* = 8.58280 (10) Å, *c* = 8.15540 (10) Å, *V* = 530.633(11) Å^3^, *Z* = 2, *ρ*_calcd_ = 1.328 g cm^−3^, and *T* = 99.9(5) K, μ(Cu Kα) = 0.835 mm^−1^. A total of 5131 reflections were measured with 2206 independent reflections (*R*_int_ = 0.0188, *R*_sigma_ = 0.0215). Final *R* indices [I>2*σ* (*I*)]: *R*_1_ = 0.0310, w*R*_2_ = 0.0860. Final R indices [all date]: *R*_1_ = 0.0314, w*R*_2_ = 0.0863, Flack parameter = 0.07(7). Largest diff. peak and hole = 0.17 and −0.20eÅ^−3^.

### 3.7. Antibacterial Activity Assay 

Compounds **1**–**21**, excepting **2**, were tested for their antibacterial activities against five pathogenic bacteria, using the method of agar filter paper diffusion. Those compounds which had an inhibition zone were tested in 96-well plates using a modification of the broth microdilution method [5]. Ampicillin and gentamicin were used as a positive control for the gram-positive and gram-negative bacteria, respectively.

### 3.8. Cytotoxicity Assay 

The cytotoxic activities of compounds **1**–**21**, excepting **2**, against human gastric cancer cells MGC803, human breast cancer cells MDA-MB-231, human prostate cancer cells C4-2B, human osteosarcoma 143B, and human hung cancer cells A549 were evaluated using the CCK-8 method [27].

## 4. Conclusions

Two new alkaloids (**1** and **2**) and one new pyrone derivative (**3**), along with eighteen known compounds (**4**–**21**) were isolated from the rice cultures of sponge-derived fungus *Penicillium* sp. SCSIO41015. The new structures were elucidated by analysis of their NMR data and HR-ESI-MS. Their absolute configurations were established by single-crystal X-ray diffraction analysis or a comparison of the experimental with the reported specific rotation values.The biosynthetic pathway of quinolactacin A has been studied, andspeculated that it is synthesized from tryptophan and isoleucine [11]. Based on the current investigation, possible biosynthetic pathways of alkaloids **1**, **2**, and **4**–**6** were speculated.Compound **2** appeared to be synthesized from tryptophan and leucine, whereas compound **4** was made from tryptophan and valine. Compounds **1**–**21**, excepting **2**, were evaluated for their cytotoxic activities againstsix humancancer cells and their antibacterial activities against five pathogenic bacteria. Only compound **16** exhibited selective inhibitory activity against the MGC803 cells with an IC_50_ value of 5.19 μM. Other compounds showed no inhibitory activities at concentrations of 5 μM in the preliminary screening. While compounds **9** and **16** displayed an antibacterial ability against *S. aureus* with MIC values of 57 and 3.75 μg/mL, respectively. Compound **18** exhibited a weak antibacterial ability against *A. baumannii*, with an MIC value of 57 μg/mL.

## Figures and Tables

**Table 1:** ^1^H NMR and ^13^C NMR data for compounds **1**–**3**.

No.	1 *^a^*		No.	2 *^a^*		No.	3 *^b^*	
	*δ*_C_	*δ*_H_		*δ*_C_	*δ*_H_		*δ*_C_	*δ*_H_
1	116.3 C		1	167.9 C		2	168.6 C	
2	151.4 C		3	52.5 CH	4.86 brd (8.4)	3	98.9 C	
3	111.4 CH	6.74 d (8.4)	3a	165.6 C		4	168.0 C	
4	135.4 CH	7.43 td (7.7, 0.7)	4a	141.2 C		5	110.0 C	
5	114.0 CH	6.60 td (7.7, 0.7)	5	117.0 CH	7.82 m	6	161.6 C	
6	131.9 CH	7.81 dd (7.7, 1.4)	6	132.6 CH	7.82 m	7	8.9 CH_3_	1.93 s
7	198.1 C		7	124.3 CH	7.49 ddd (7.7, 4.9, 2.8)	8	10.0 CH_3_	2.01 s
8a 8b	40.2 CH_2_	3.44 dd (17.5, 7.0) 3.40 dd (17.5, 5.6)	8	125.9 CH	8.26 d (7.7)	9	43.8 CH	2.95 qui (7.0)
9	47.9 CH	4.76 td (7.0, 5.6)	8a	128.1 C		10	70.5 CH	3.94 qui (6.5)
10	172.2 C		9	171.8 C		11	21.2 CH_3_	1.27 d (6.5)
11	29.0 CH_3_	2.85 d (4.9)	9a	109.3 C		12	14.8 CH_3_	1.17 d (7.0)
12	169.2 C		10	42.3 CH_2_	1.79–1.88 m 1.37 ddd (12.6, 9.1, 2.8)			
13	22.3 CH_3_	1.82 s	11	24.7 CH	1.79–1.88 m			
14	52.0 CH_3_	3.61 s	12	23.5 CH_3_	0.83 d (6.3)			
NH-9		8.23 d (7.7)	13	21.5 CH_3_	1.01 d (6.3)			
NH-11		8.59 dd (9.1, 4.2)	14	35.8 CH_3_	3.79 s			
			NH		8.27 m			

*^a^* Measured at 700, 175 MHz NMR in DMSO-*d*_6_; *^b^* Measuredat 500, 125 MHz NMR in MeOH-*d*_4_.

## Supplementary Materials

The following are available online at https://www.mdpi.com/1660-3397/17/7/398/s1, The ITS sequences data of *Penicillium* sp. SCSIO41015, the 1D and 2D NMR spectra, HR–ESI–MS for the new compounds **1**–**3**, and X-ray crystallographic files of compounds **2** and **3** (in CIF format).
