# Supplementary material for: New Alkaloids and Polyketides from the Marine Sponge-Derived Fungus Penicillium sp. SCSIO41015
Source: Mar Drugs. 2019 Jul 5;17(7):398. doi: 10.3390/md17070398 (PMC6669684; doi:10.3390/md17070398)
Supplement: Supplementary file 1 [file marinedrugs-17-00398-s001.pdf]

New Alkaloids and Polyketides from the Marine Sponge-Derived Fungus *Penicillium* sp. SCSIO41015

Xiaoyan Pang[1,2,3], Guodi Cai[4], Xiuping Lin[2], Limbadri Salendra[2], Xuefeng Zhou[2], Bin Yang[2], Junjian Wang[4], Junfeng Wang[2*], Shihai Xu[1*], Yonghong Liu[2*]

[1] College of Chemistry and Materials Science, Jinan University, Guangzhou 510632, China; luckygirlpxy@163.com (X.P.)

[2] CAS Key Laboratory of Tropical Marine Bio-resources and Ecology/Guangdong Key Laboratory of Marine Materia Medica, South China Sea Institute of Oceanology, Chinese Academy of Sciences, Guangzhou 510301, China; raj.badri202@gmail.com (L.S.); xiupinglin@hotmail.com (X.L.); xfzhou@scsio.ac.cn (X.Z.); yangbin@scsio.ac.cn (B.Y.);

[3] College of Pharmacy, Jinan University, Guangzhou 510632, China;

[4] School of Pharmaceutical Sciences, Sun Yat-Sen University, Guangzhou 510006, China; caigd3@mail2.sysu.edu.cn (G.C.); wangjj87@mail.sysu.edu.cn (J.J.W.)

* Correspondence: wangjunfeng@scsio.ac.cn (J.F.W.); txush@jnu.edu.cn (S.X.); yonghongliu@scsio.ac.cn (Y.L.); Tel: +86-020-8902-3174 (J.F.W.); +86-020-85220223 (S.X.); +86-020-8902-3244 (Y.L.)

List of Supporting Information

The ITS sequences data of *Penicillium* sp. SCSIO41015

Figure S1. The possible biosynthesis pathway of alkaloids.

Figure S2. The preliminary screening results of the antibacterial assay.

Table S1. The MIC values of compounds with antibacterial activities in preliminary screening.

Figure S3. ^{1}H NMR spectrum of **1** in CD_3OD.

Figure S4. ^{13}C NMR spectrum of **1** in CD_3OD.

Figure S5. DEPT NMR spectrum of **1** in CD_3OD.

Figure S6. ^{1}H NMR spectrum of **1** in DMSO-d_6.

Figure S7. ^{13}C NMR spectrum of **1** in DMSO-d_6.

Figure S8. ^{1}H-^{1}H COSY spectrum of **1** in DMSO-d_6.

Figure S9. HSQC spectrum of **1** in DMSO-d_6.

Figure S10.HMBC spectrum of **1** in DMSO-d_6.

Figure S11. HRESIMS spectrum of **1**.

Figure S12. ^{1}H NMR spectrum of **2** in DMSO-d_6.

Figure S13. ^{13}C NMR spectrum of **2** in DMSO-d_6.

Figure S14. DEPT NMR spectrum of **2** in DMSO-d_6.

Figure S15. ^{1}H-^{1}H COSY spectrum of **2** in DMSO-d_6.

Figure S16. HSQC spectrum of **2** in DMSO-d_6.

Figure S17. HMBC spectrum of **2** in DMSO-d_6.

Figure S18. HRESIMS spectrum of **2**.

Figure S19. ^{1}H NMR spectrum of **3** in CD_3OD.

Figure S20. ^{13}C NMR spectrum of **3** in CD_3OD.

Figure S21. ^{1}H-^{1}H COSY spectrum of **3** in CD_3OD.

Figure S22. HSQC spectrum of **3** in CD_3OD.

Figure S23. HMBC spectrum of **3** in CD_3OD.

Figure S24. HRESIMS spectrum of **3**.

The ITS sequences data of *Penicillium* sp. SCSIO41015

CTCGGGGCCACCTCCCACCCGTGTTGCCCGAACCTATGTTGCCTCGGCGGGCCCCGCG
CCCGCCGACGGCCCCCCTGAACGCTGTCTGAAGTTGCAGTCTGAGACCTATAACGAA
ATTAGTTAAAACTTTCAACAACGGATCTCTTGGTTCCGGCATCGATGAAGAACGCAGC
GAAATGCGATAACTAATGTGAATTGCAGAATTCAGTGAATCATCGAGTCTTTGAACG
CACATTGCGCCCTCTGGTATTCCGGAGGGCATGCCTGTCCGAGCGTCATTGCTGCCCT
CAAGCCCGGCTTGTGTGTTGGGCCCCGTCCCCCCCGCCGGGGGGACGGGCCCGAAAG
GCAGCGGCGGCACCGCGTCCGGTCCTCGAGCGTATGGGGCTTCGTCACCCGCTCTAGT
AGGCCCGGCCGGCGCCAGCCGACCCCCAACCTTTAATTATCTCAGGTTGACCTCGGAT
CAGGTAGGGATACCCGCTGAACTTAAGCATATCAATAAGCGGAGGAA

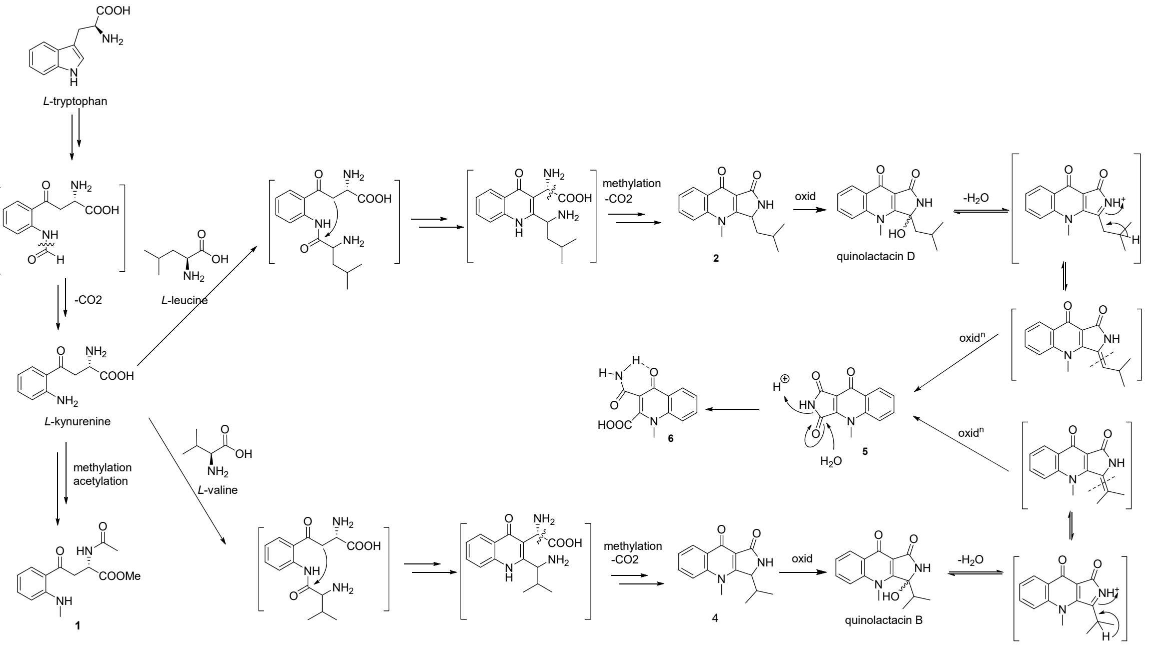

Figure S1. The possible biosynthesis pathway of alkaloids speculated referring to the references of *Org. Biomol. Chem.* **2006**,*4*, 1512-1519 and *J. Antibiot.* **2006**, *59*, 418-427.

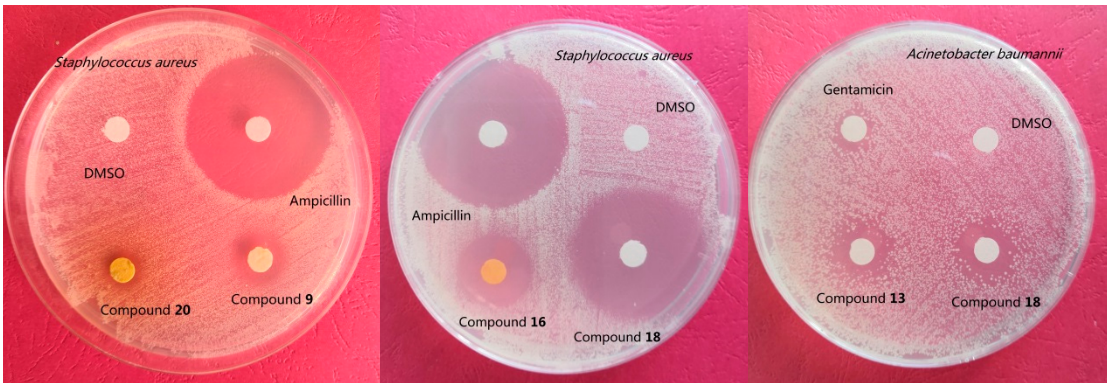

Figure S2. The preliminary screening results of the antibacterial assay to compounds **1**–**21** except **2**. In the assay all the tested compounds with 100 μg/disc (the diameter of the disc is 6 mm), ampicillin with 6.4 μg/disc, and gentamicin with 3.2 μg/disc.

Table S1 The MIC values of compounds with antibacterial activities in preliminary screening.

Compounds	*Staphyloccocus aureus* ATCC 29213	*Acinetobacter baumannii* ATCC 19606
**9**	57 μg/mL	–
**21**	227 μg/mL	–
**16**	3.75 μg/mL	–
**18**	>454 μg/mL	57 μg/mL
**13**	–	>454 μg/mL
Ampicillin	0.45 μg/mL	–
Gentamicin	–	0.9 μg/mL

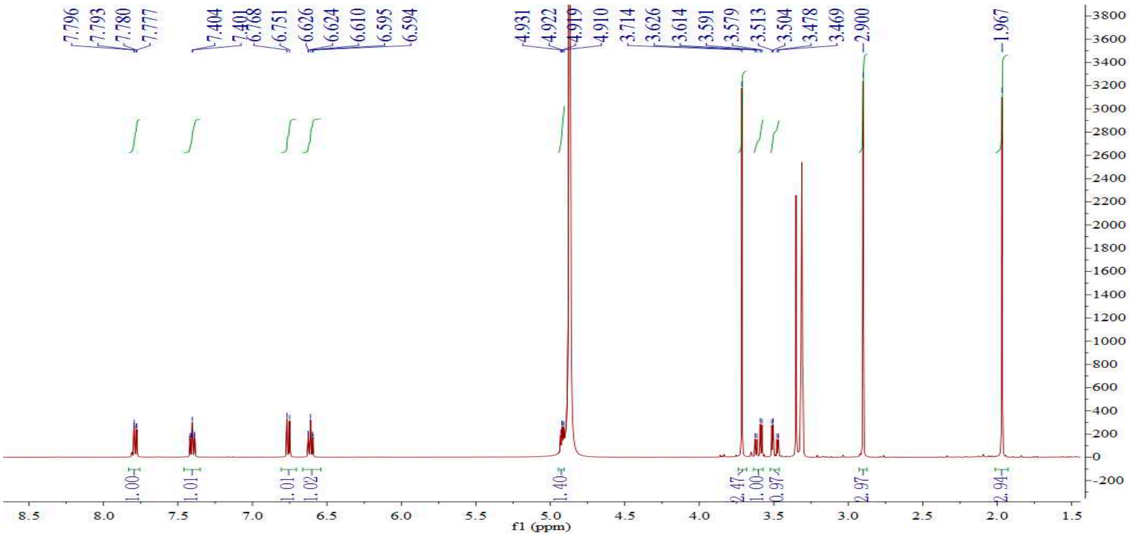

Figure S3. ^{1}H NMR spectrum of **1** in CD_3OD.

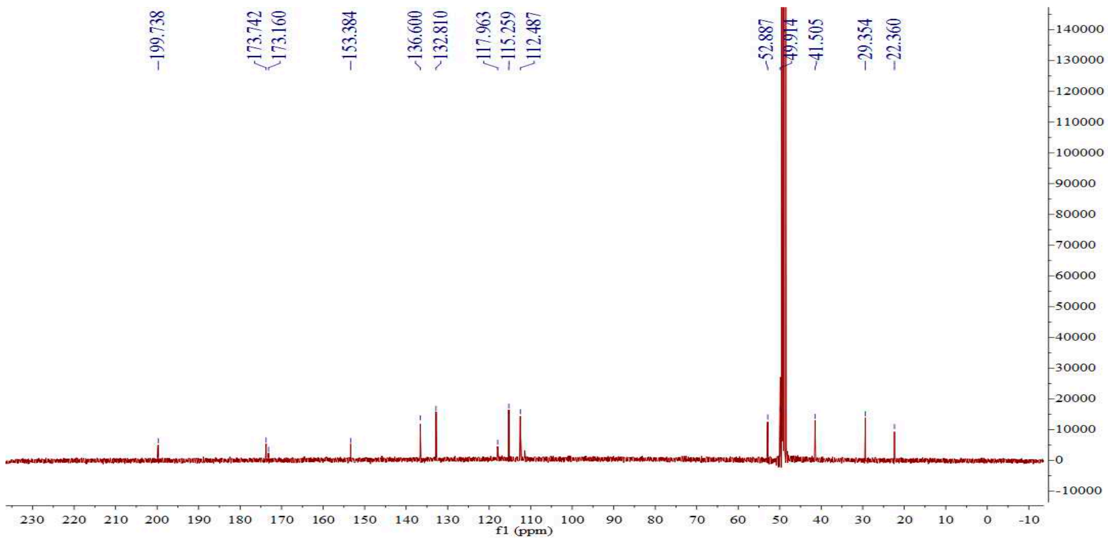

Figure S4. ^{13}C NMR spectrum of **1** in CD_3OD.

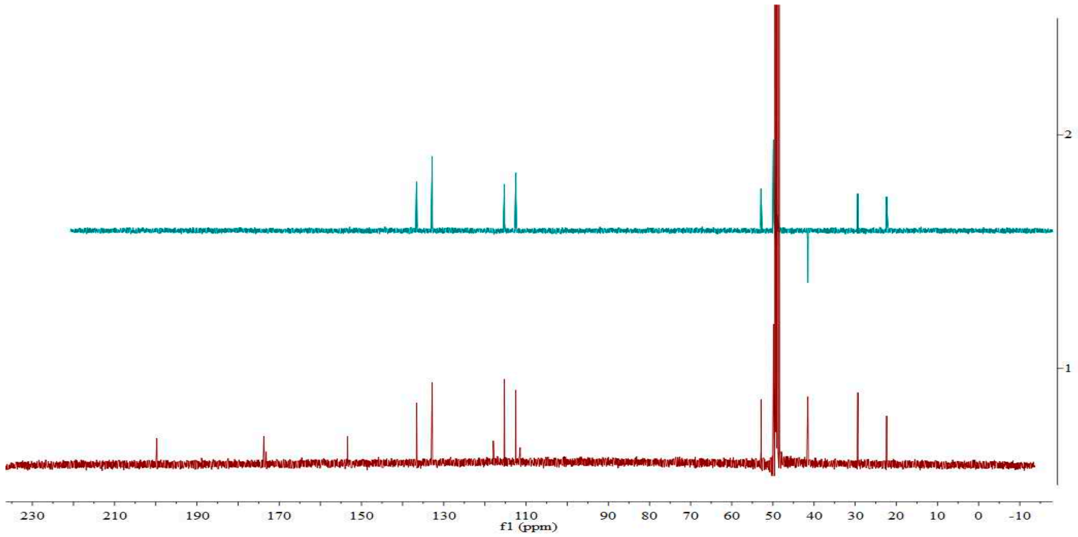

Figure S5. DEPT NMR spectrum of **1** in CD_3OD.

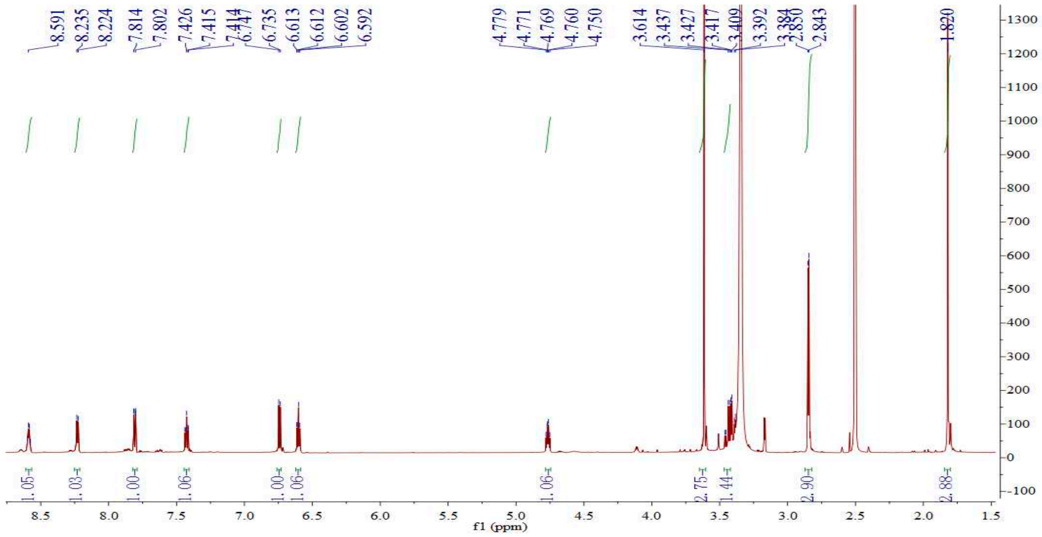

Figure S6. ^{1}H NMR spectrum of **1** in DMSO-d_6.

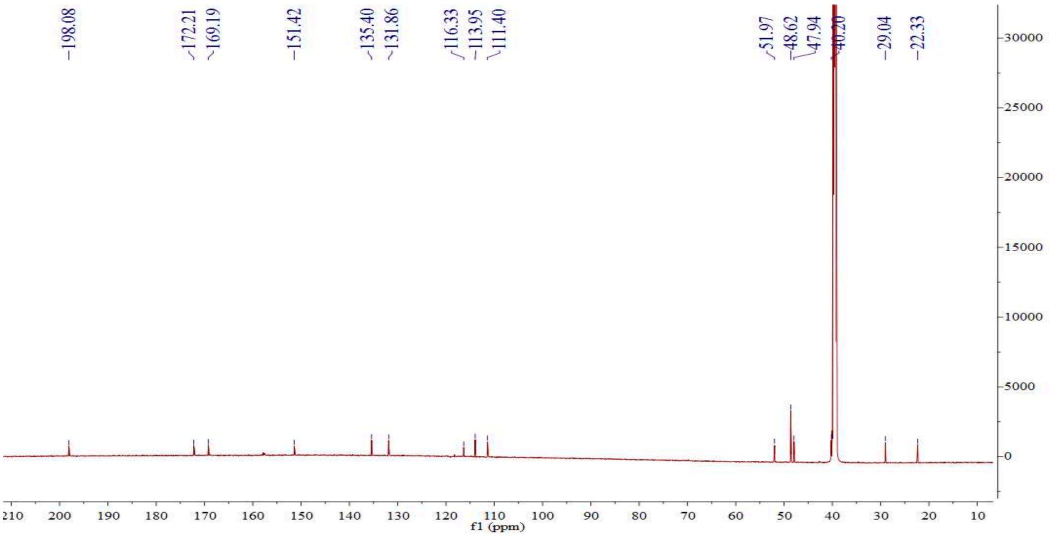

Figure S7. ^{13}C NMR spectrum of **1** in DMSO-d_6.

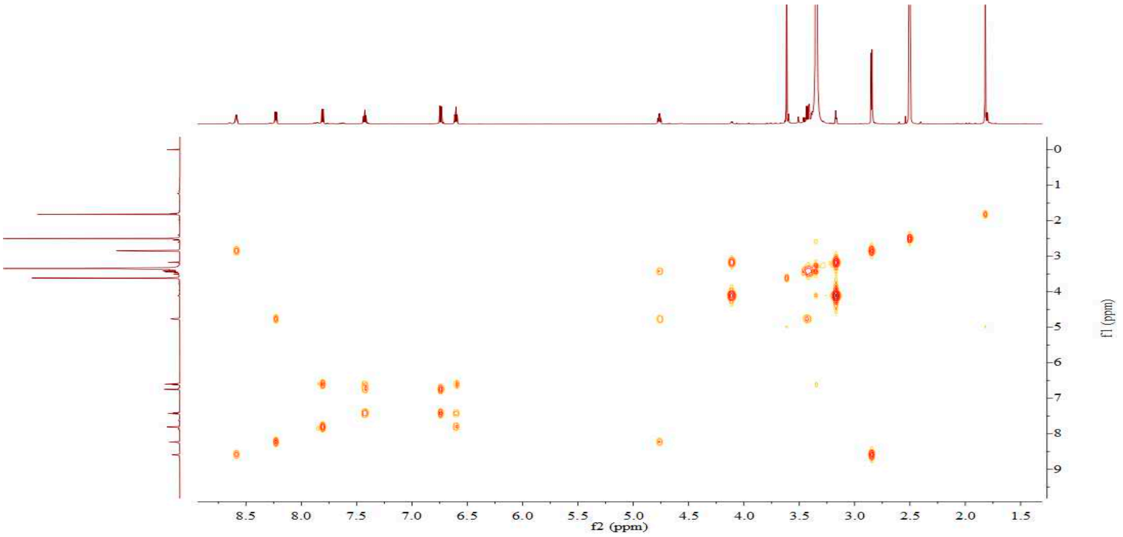

Figure S8. ^{1}H-^{1}H COSY spectrum of **1** in DMSO-d_6.

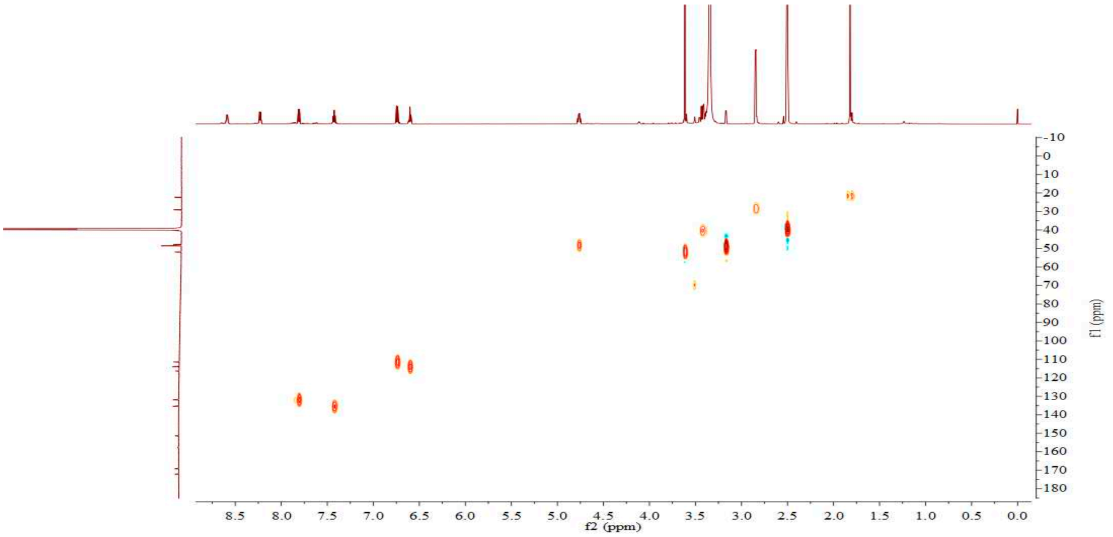

Figure S9. HSQC spectrum of **1** in DMSO-d_6.

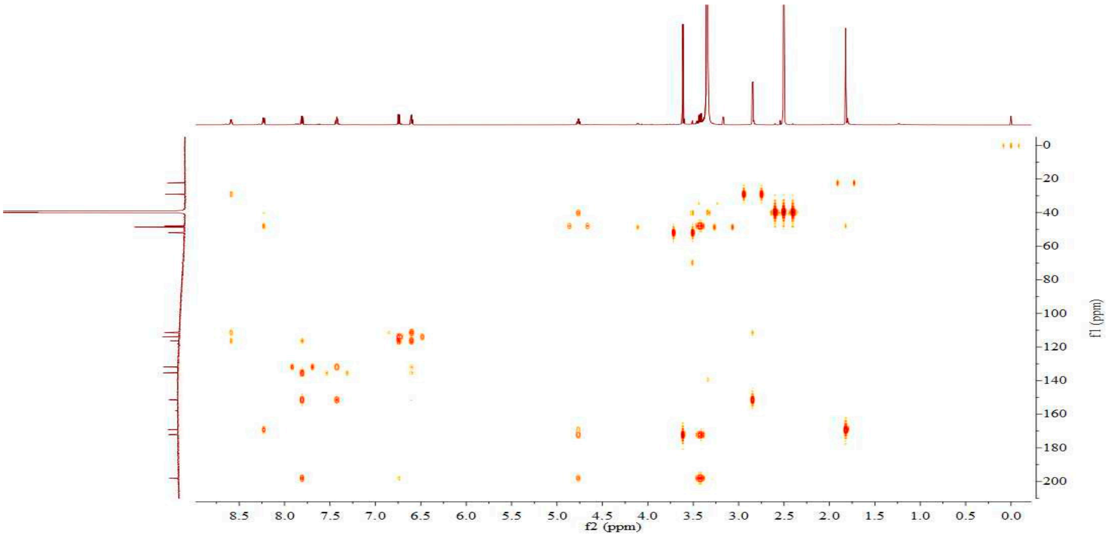

Figure S10. HMBC spectrum of **1** in DMSO-d_6.

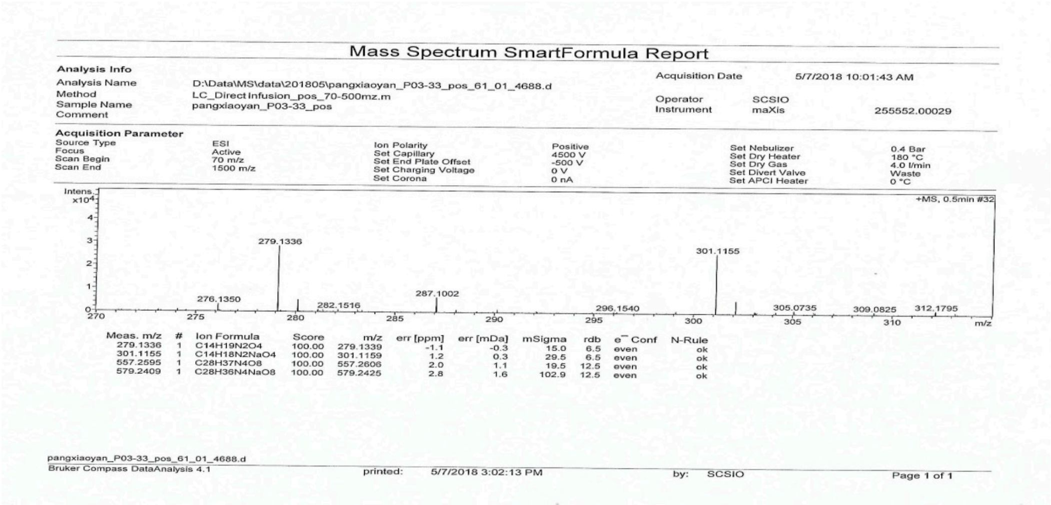
Mass Spectrum SmartFormula Report

Analysis Info
Analysis Name D:\Data\MS\data\201805\pangxiaoyan_P03-33_pos_61_01_4688.d
Method LC_Direct Infusion_pos_70-500mz.m
Sample Name pangxiaoyan_P03-33_pos
Comment
Acquisition Date 5/7/2018 10:01:43 AM
Operator SCSIO
Instrument maXis 255552.00029

Acquisition Parameter

Source Type	ESI	Ion Polarity	Positive	Set Nebulizer	0.4 Bar
Focus	Active	Set Capillary	4500 V	Set Dry Heater	180 °C
Scan Begin	70 m/z	Set End Plate Offset	-500 V	Set Dry Gas	4.0 l/min
Scan End	1500 m/z	Set Charging Voltage	0 V	Set Divert Valve	Waste
		Set Corona	0 nA	Set APCI Heater	0 °C

Meas. m/z	#	Ion Formula	Score	m/z	err [ppm]	err [mDa]	mSigma	rdb	e⁻ Conf	N-Rule
279.1336	1	C14H19N2O4	100.00	279.1339	-1.1	-0.3	15.0	6.5	even	ok
301.1155	1	C14H18N2NaO4	100.00	301.1159	1.2	0.3	29.5	6.5	even	ok
557.2595	1	C28H37N4O8	100.00	557.2606	2.0	1.1	19.5	12.5	even	ok
579.2409	1	C28H36N4NaO8	100.00	579.2425	2.8	1.6	102.9	12.5	even	ok

pangxiaoyan_P03-33_pos_61_01_4688.d
Bruker Compass DataAnalysis 4.1 printed: 5/7/2018 3:02:13 PM by: SCSIO Page 1 of 1

Figure S11. HRESIMS spectrum of **1**.

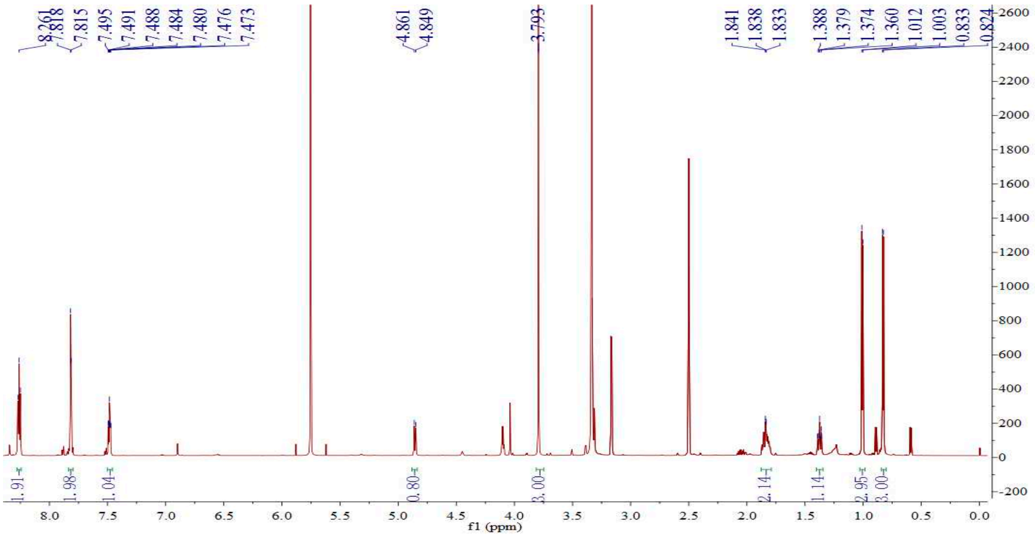

Figure S12. ^{1}H NMR spectrum of **2** in DMSO-d_6.

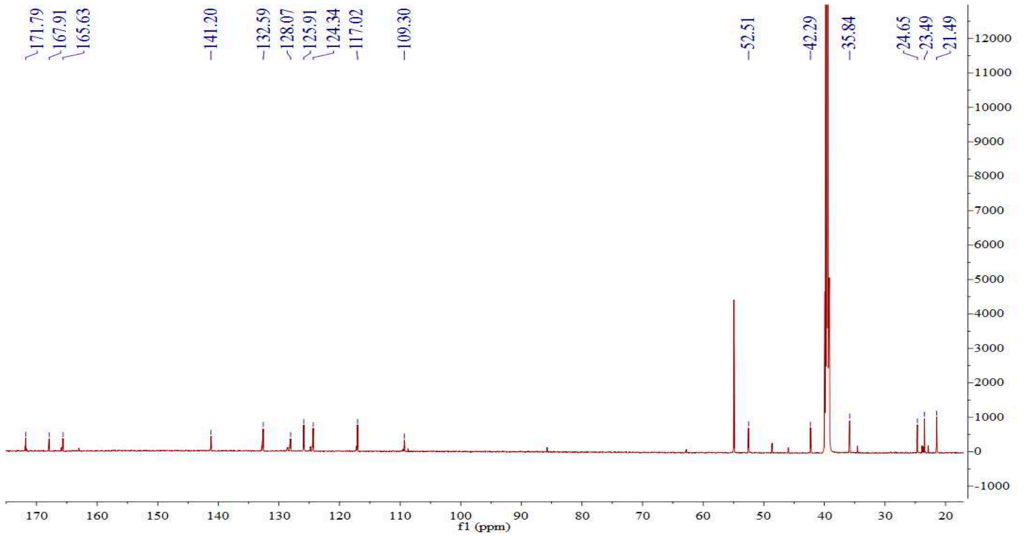

Figure S13. ^{13}C NMR spectrum of **2** inDMSO-*d*6.

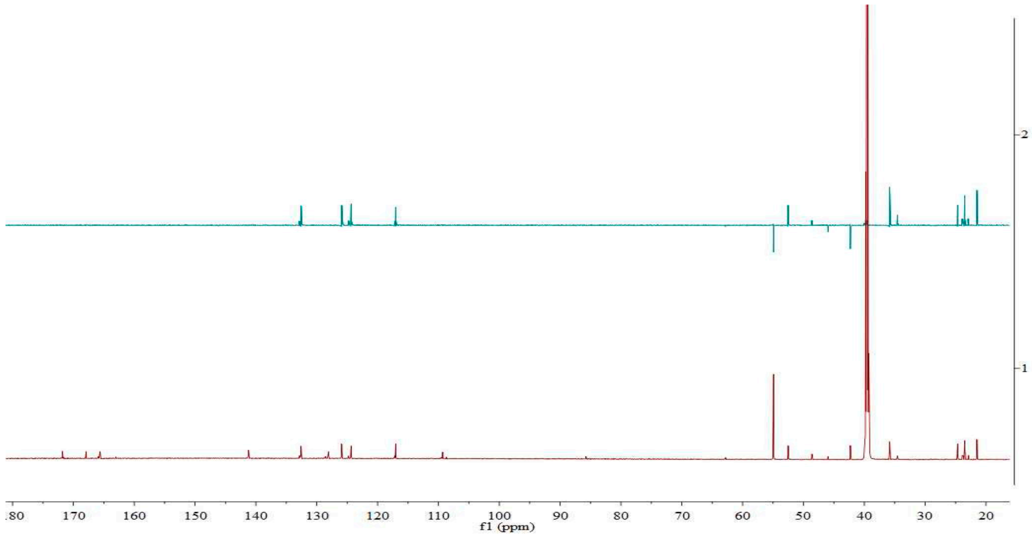

Figure S14. DEPT NMR spectrum of **2** in DMSO-*d*6.

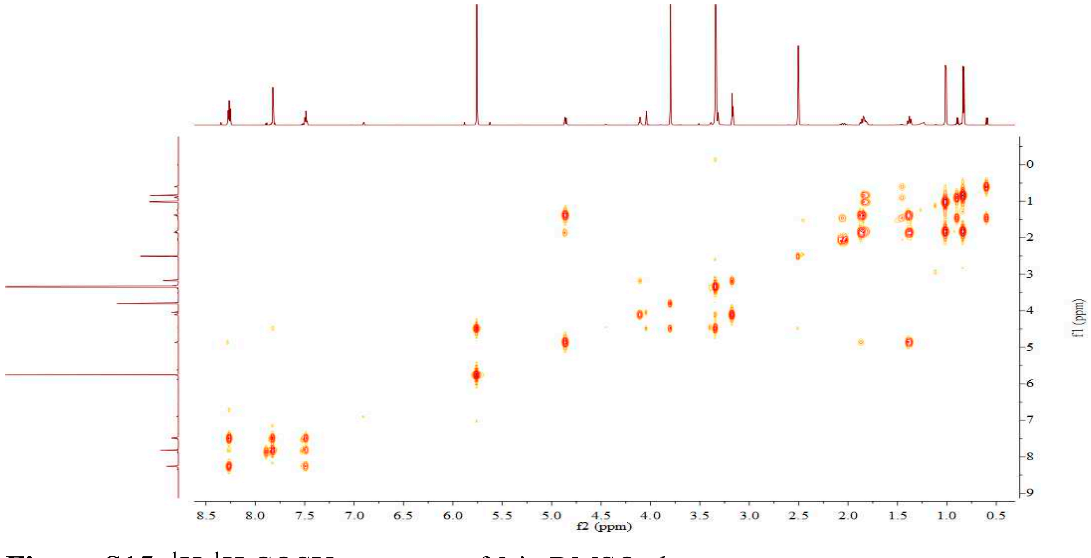

Figure S15. ^{1}H-^{1}H COSY spectrum of **2** in DMSO-*d*6.

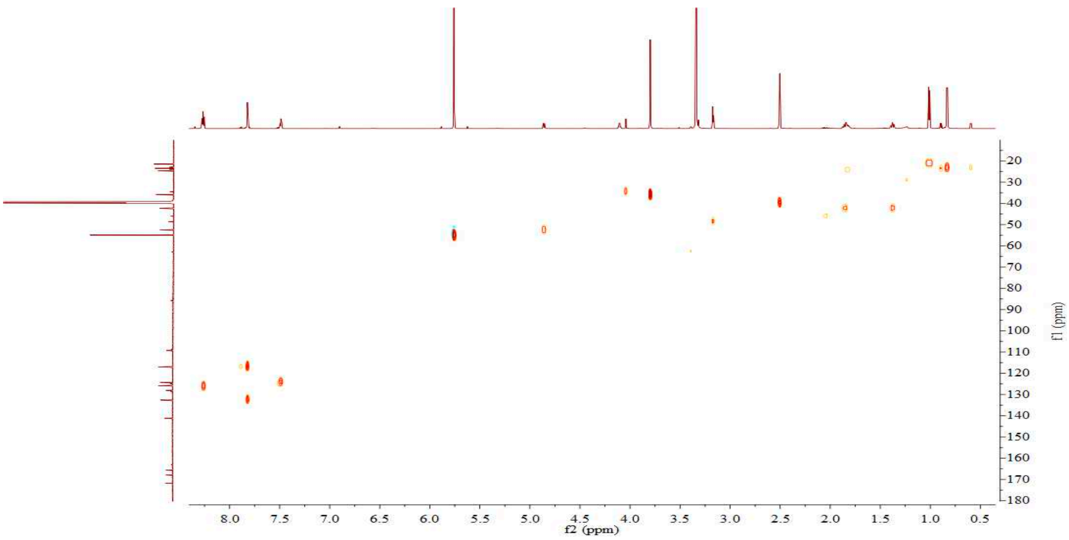

Figure S16. HSQC spectrum of **2** in DMSO-d_6.

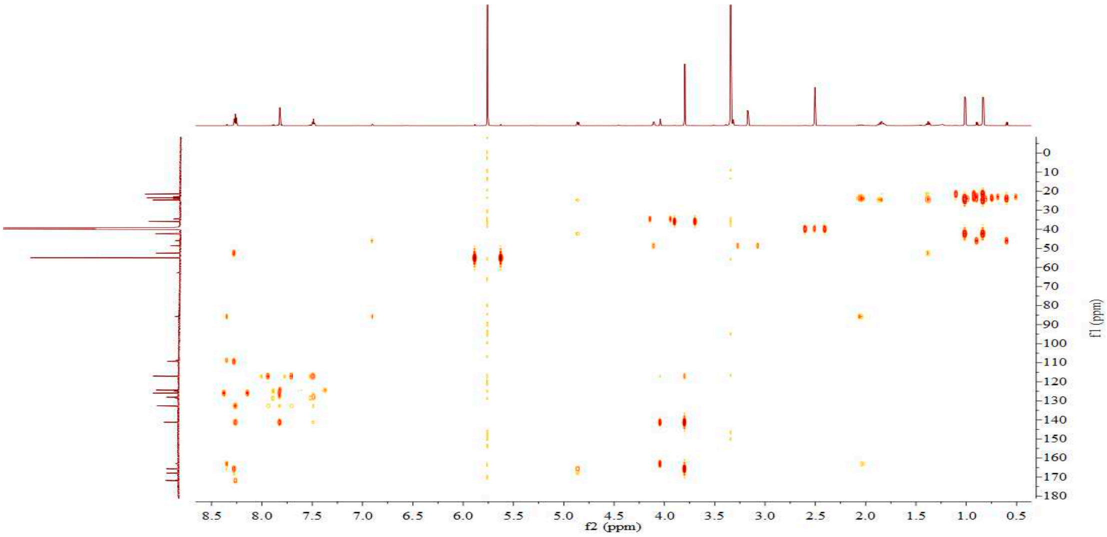

Figure S17. HMBC spectrum of **2** in DMSO-d_6.

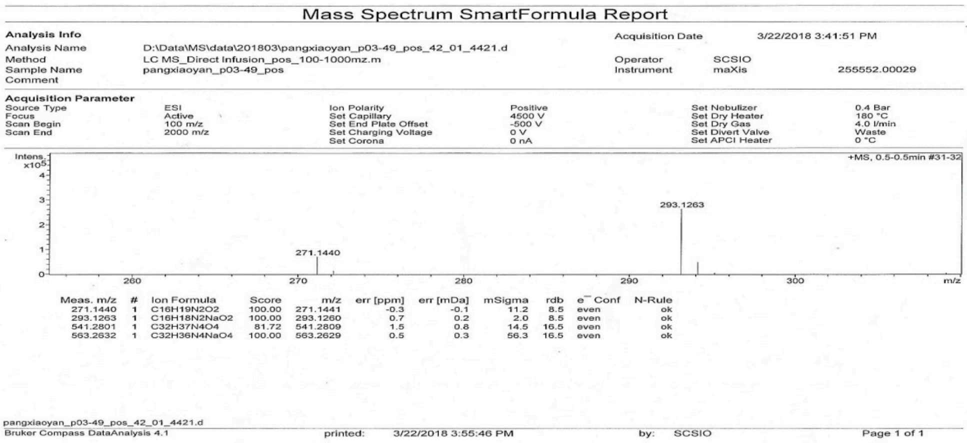

Mass Spectrum SmartFormula Report

Analysis Info

Analysis Name	D:\Data\MS\data\201803\pangxiaoyan_p03-49_pos_42_01_4421.d	Acquisition Date	3/22/2018 3:41:51 PM
Method	LC MS_Direct Infusion_pos_100-1000mz.m	Operator	SCSIO
Sample Name	pangxiaoyan_p03-49_pos	Instrument	maXis 255552.00029
Comment			

Acquisition Parameter

Source Type	ESI	Ion Polarity	Positive	Set Nebulizer	0.4 Bar
Focus	Active	Set Capillary	4500 V	Set Dry Heater	180 °C
Scan Begin	100 m/z	Set End Plate Offset	-500 V	Set Dry Gas	4.0 l/min
Scan End	2000 m/z	Set Charging Voltage	0 V	Set Divert Valve	Waste
		Set Corona	0 nA	Set APCI Heater	0 °C

Meas. m/z	#	Ion Formula	Score	m/z	err [ppm]	err [mDa]	mSigma	rdb	e⁻ Conf	N-Rule
271.1440	1	$C_{16}H_{19}N_2O_2$	100.00	271.1441	-0.3	-0.1	11.2	8.5	even	ok
293.1263	1	$C_{16}H_{18}N_2NaO_2$	100.00	293.1260	0.7	0.2	2.0	8.5	even	ok
541.2801	1	$C_{32}H_{37}N_4O_4$	81.72	541.2809	1.5	0.8	14.5	16.5	even	ok
563.2632	1	$C_{32}H_{36}N_4NaO_4$	100.00	563.2629	0.5	0.3	56.3	16.5	even	ok

pangxiaoyan_p03-49_pos_42_01_4421.d
Bruker Compass DataAnalysis 4.1 printed: 3/22/2018 3:55:46 PM by: SCSIO Page 1 of 1

Figure S18. HRESIMS spectrum of **2**.

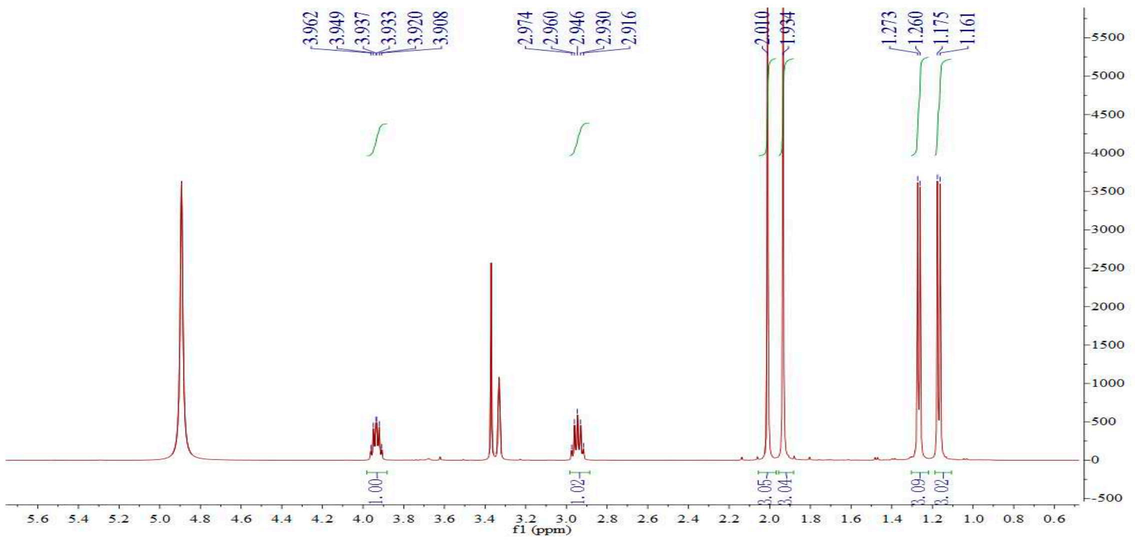

Figure S19. ^{1}H NMR spectrum of **3** in CD_3OD.

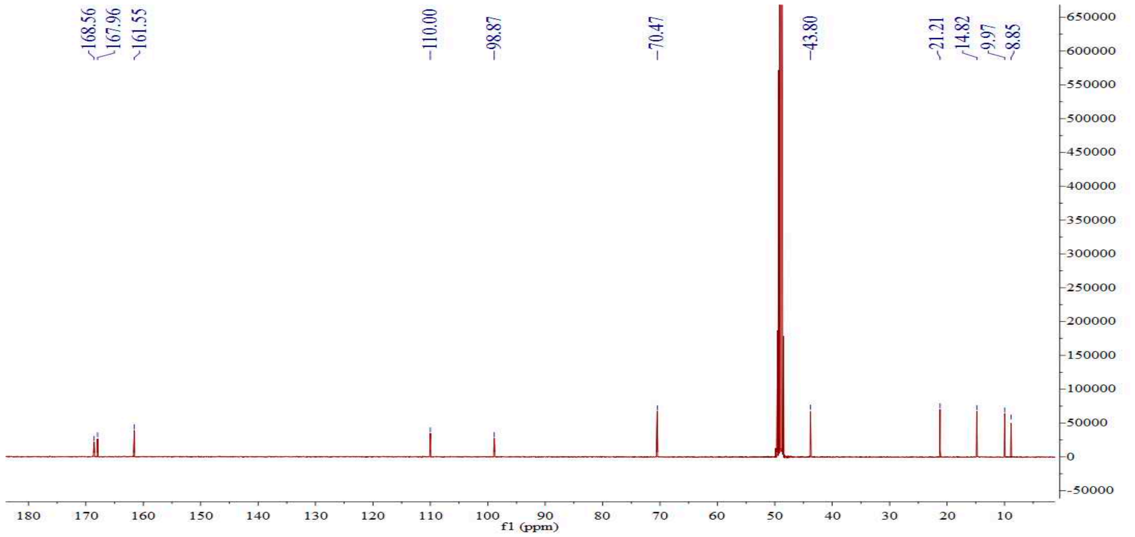

Figure S20. ^{13}C NMR spectrum of **3** in CD_3OD.

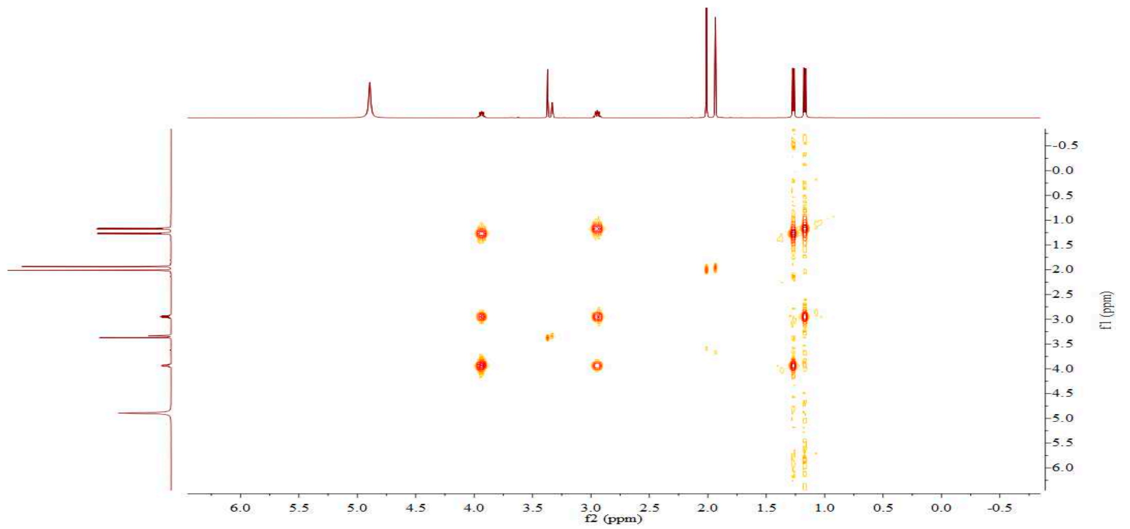

Figure S21. ^{1}H-^{1}H COSY spectrum of **3** in CD_3OD.

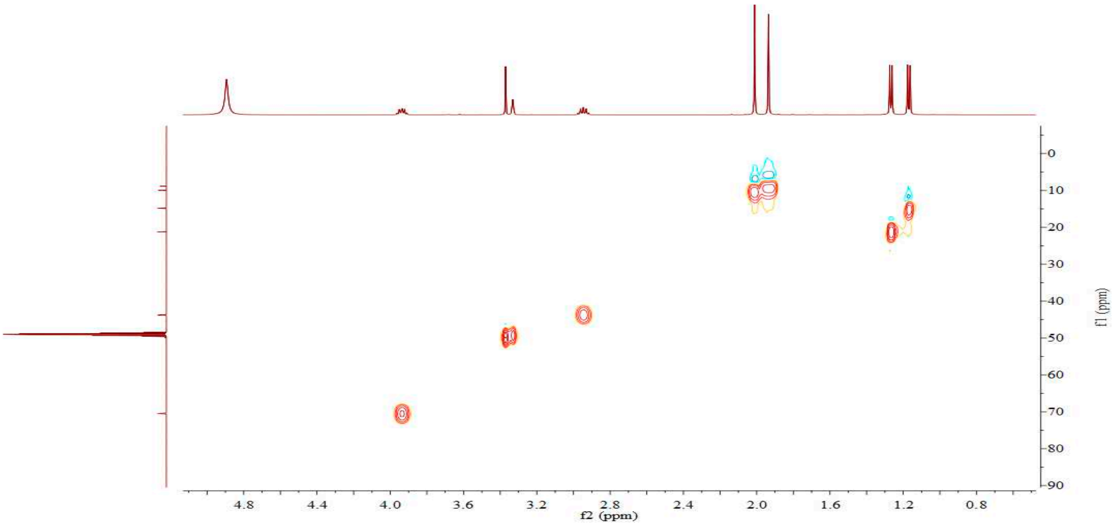

Figure S22. HSQC spectrum of **3** in CD_3OD.

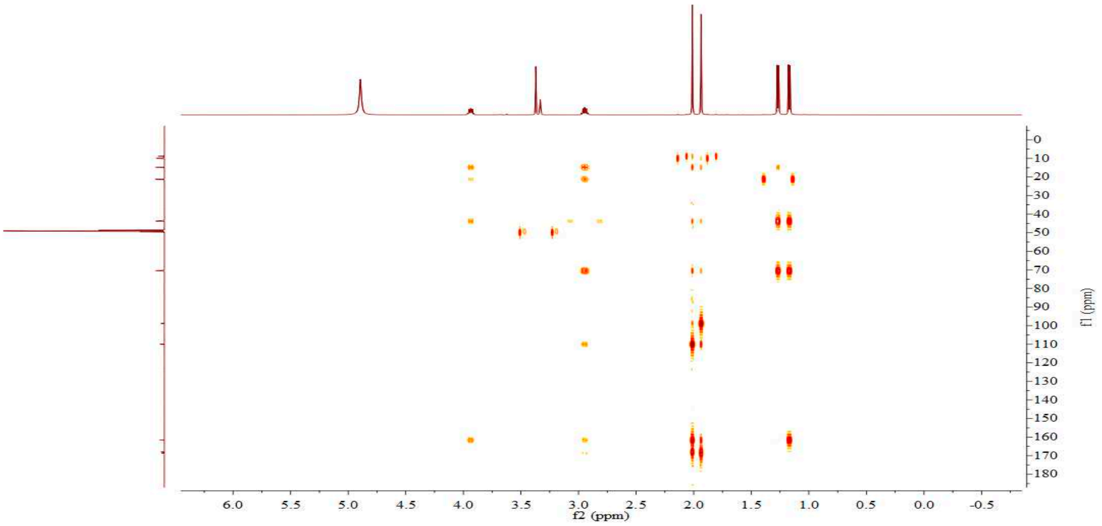

Figure S23. HMBC spectrum of **3** in CD_3OD.

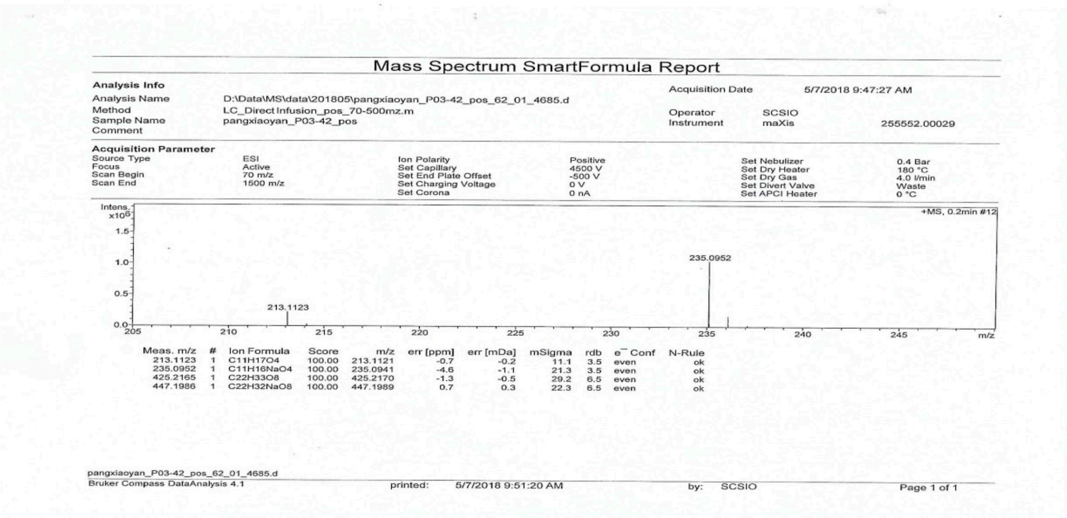

Mass Spectrum SmartFormula Report

Analysis Info		Acquisition Date	5/7/2018 9:47:27 AM
Analysis Name	D:\Data\MS\data\201805\pangxiaoyan_P03-42_pos_62_01_4685.d		
Method	LC_Direct Infusion_pos_70-500mz.m	Operator	SCSIO
Sample Name	pangxiaoyan_P03-42_pos	Instrument	maXis 255552.00029
Comment			

Acquisition Parameter					
Source Type	ESI	Ion Polarity	Positive	Set Nebulizer	0.4 Bar
Focus	Active	Set Capillary	4500 V	Set Dry Heater	180 °C
Scan Begin	70 m/z	Set End Plate Offset	-500 V	Set Dry Gas	4.0 l/min
Scan End	1500 m/z	Set Charging Voltage	0 V	Set Divert Valve	Waste
		Set Corona	0 nA	Set APCI Heater	0 °C

Meas. m/z	#	Ion Formula	Score	m/z	err [ppm]	err [mDa]	mSigma	rdb	e⁻ Conf	N-Rule
213.1123	1	C11H17O4	100.00	213.1121	-0.7	-0.2	11.1	3.5	even	ok
235.0952	1	C11H16NaO4	100.00	235.0941	-4.6	-1.1	21.3	3.5	even	ok
425.2165	1	C22H33O8	100.00	425.2170	-1.3	-0.5	29.2	6.5	even	ok
447.1986	1	C22H32NaO8	100.00	447.1989	0.7	0.3	22.3	6.5	even	ok

pangxiaoyan_P03-42_pos_62_01_4685.d
Bruker Compass DataAnalysis 4.1 printed: 5/7/2018 9:51:20 AM by: SCSIO Page 1 of 1

Figure S24. HRESIMS spectrum of **3**.
